# New conception for the development of hypertension in preeclampsia

**DOI:** 10.18632/oncotarget.13410

**Published:** 2016-11-16

**Authors:** Qinqin Gao, Jiaqi Tang, Na Li, Xiuwen Zhou, Xiaolin Zhu, Weisheng Li, Bailin Liu, Xueqin Feng, Jianying Tao, Bing Han, Hong Zhang, Miao Sun, Zhice Xu

**Affiliations:** ^1^ Institute for Fetology, First Affiliated Hospital of Soochow University, Suzhou, China; ^2^ Center for Perinatal Biology, Loma Linda University, California, USA; ^3^ Department of Obstetrics and Gynecology, First and Second Affiliated Hospital of Soochow University, Municipal Hospital, Suzhou, China

**Keywords:** nitric oxide, cGMP/sGC pathway, vascular dysfunction, placenta, preeclampsia, Pathology Section

## Abstract

Placental vascular dysfunction was suggested to be critical for placental ischemia-initiated hypertension in preeclampsia, although the contributions of endothelium involved are unclear. The present study found, unlike non-placental vessels, acetylcholine showed no vasodilatation effect on placental vessels, indicating that endothelial-derived nitric oxide (NO) was extremely weak in placental vessels. Placental vascular responses to exogenous NO from sodium nitroprusside (SNP) were significantly different from non-placental vessels. These results were further confirmed in sheep, and rat vessels. In preeclamptic placental vessels, acetylcholine also showed no vasodilatation effects, while vascular responses to SNP were suppressed, associated with impaired cGMP/sGC pathway in vascular smooth muscle cells (VSMCs). The current theory on placental ischemia-initiated hypertension in preeclampsia focused on changes in placental vascular functions, including endothelial dysfunction. This study found the placental endothelium contributed very poorly to vasodilatation, and altered vascular functions in preeclampsia mainly occurred in VSMCs instead of endothelial cells. The findings contribute importantly to understanding the special feature of placental vascular functions and its pathophysiological changes in the development of hypertension in preeclampsia.

## INTRODUCTION

A fascinating clinical problem in human pregnancy has puzzled scientists and doctors for long history: why many normal blood pressure women show hypertension during pregnancy and this phenomenon disappears soon or later following the end of pregnancy in most cases? Although numerous factors including immunological, oxidative stress, environmental, and genetic factors have been implicated in development of hypertension in preeclampsia [1–3], inadequate blood flow or ischemia in the placenta is an important initiating event in preeclampsia [4, 5]. Placental insufficient trophoblastic invasion of uterine spiral arteries and shallow placentation have been considered structural cause for inducing preeclampsia [5–7]. However, placental vascular functional changes in development of hypertension in preeclampsia are unclear.

Vascular dysfunction can be divided into disability of relaxation and abnormal constriction, either or both of them are fundamental basis for a variety of vascular diseases, including hypertension during pregnancy. In preeclampsia, vascular dysfunction includes two parts: the placental side and maternal side, while endothelial dysfunction is a key issue at both sides [8–10]. This study used a large number of human placental and non-placental vessel samples from normal and preeclamptic pregnancies, as well as animal placental and non-placental vessels, to reveal the placental vascular functional characteristics and changes under physiological and preeclamptic conditions, and determine possible contributions of altered placental vascular functions in development of hypertension in preeclampsia.

## RESULTS

### Endothelium-dependent vasodilatation in placental vs. non-placental vessels

**Figure 1:**
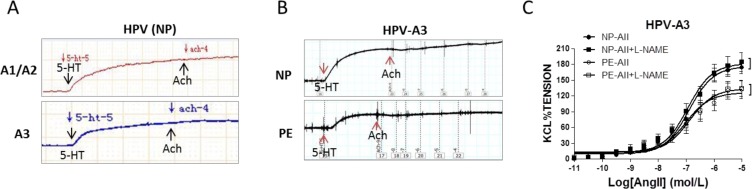
Endothelium-dependent vasodilatation in human placenta (**A**), and (**B**), Acetylcholine (Ach) exhibited no vasodilatation following 5-HT-produced vasoconstriction in placental vessels from normal pregnancy (NP) and preeclampsia (PE) (N=52, n=124 for NP; N=37, n=93 for PE). (**C**), Pre-treated with L-NAME did not increase angiotensin II (AII)-induced vasoconstriction in NP and PE placental vessels (N=14, n=16 for NP; N=13, n=14 for PE; P>0.05). HPV-A1/A2, first-, second-order branch of blood vessels in placenta; HPV-A3, the branch of the main stem villous arteries (micro-vessels with diameter around 150um); 5-HT, serotonin; L-NAME, NG-Nitro-L-arginine Methyl Ester (a nitric oxide-synthase inhibitor). Error bars denote s.e.m. *, P<0.05; **, P<0.01. N, number of participants; n, number of vessel rings.

Acetylcholine (Ach) did not show significant dilation in human placental vessels from normal (NP) and preeclamptic pregnancies (PE) (Figure 1A and 1B, Supplementary Figure 1a and Supplementary Table 3 online), while exhibited reliable endothelium-dependent vasodilatation in sheep carotid artery, rat mesenteric artery and thoracic aorta (Supplementary Figure 1b online). In the presence of L-NAME (NG-Nitro-L-arginine Methyl Ester, a nitric oxide-synthase inhibitor), angiotensin II (AII)-induced vasoconstriction was not significantly increased in placental vessels in both the NP and PE group (Figure 1C). Pre-treatment the rings with L-NAME significantly inhibited AII-induced dose-dependent vasoconstriction in rat mesenteric arteries (Supplementary Figure 1c online).

### Nitric oxide system in human placenta

**Figure 2:**
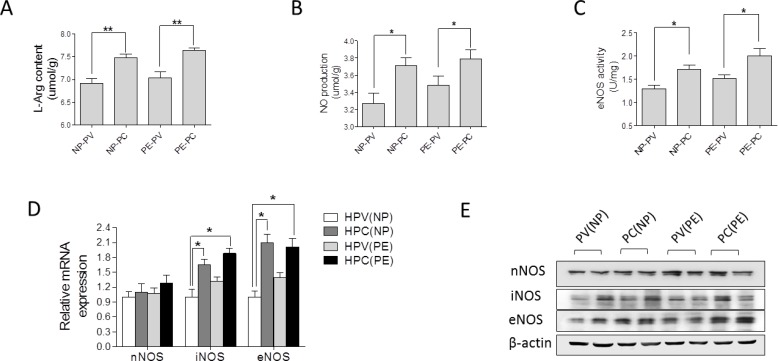
Nitric oxide (NO) system in human placenta (**A**)-(**E**), L-Arg content, NO production, eNOS enzymatic activity, mRNA and protein levels of nNOS, iNOS, and eNOS in human placental vessels (HPV) and tissue (HPC) from the NP and PE pregnancies (N=15/group). NP, normal pregnancy; PE, preeclampsia; L-Arg, L-Argnine; PV, placental vessels; PC, placental tissue; eNOS, endothelial nitric oxide synthase; nNOS, neuronal nitric oxide synthase; iNOS, inducible nitric oxide synthase. Error bars denote s.e.m. *, P<0.05; **, P<0.01. N, number of participants.

In placental tissue, L-Argnine (L-Arg) content and nitric oxide (NO) production, as well as eNOS (endothelial nitric oxide synthase) enzymatic activity were significantly higher than that in placental vessels in both the NP and PE group (Figure 2A-2C). The mRNA and protein of iNOS (inducible nitric oxide synthase) and eNOS, not nNOS (neuronal nitric oxide synthase), were markedly higher in placental tissue than that in placental vessels, regardless of normal pregnancy and preeclampsia (Figure 2D and 2E, Supplementary Figure 2 online).

### Sodium nitroprusside (SNP)-induced relaxation in placental vessels

**Figure 3:**
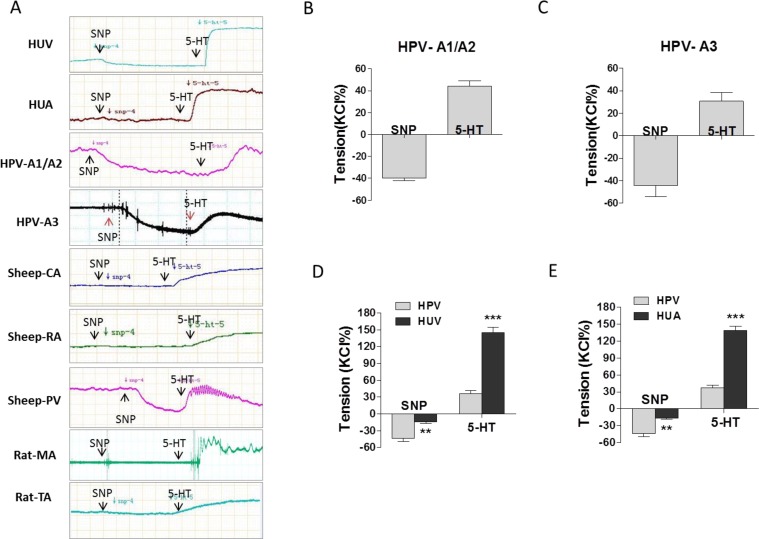
The effect of SNP on placental and non-placental vessels (**A**), representative images of SNP reduced vessel basal tension, followed by 5-HT-stimulated maximum vasoconstriction, in human umbilical vein (HUV), umbilical artery (HUA), human placental vessels (HPV-A1/A2, HPV-A3), fetal sheep carotid artery (CA), fetal sheep renal artery (RA), fetal sheep placental vessels (PV), rat mesenteric artery (MA), and rat thoracic aorta (TA). (**B**)-(**E**), SNP reduced basal tension and 5-HT-stimulated maximum vasoconstriction in HPV-A1/A2 (N=31, n=76), HPV-A3 (N=35, n=41), HUV (N=31, n=76) *vs.* HPV, and HUA (N=21, n=46) *vs.* HPV. HPV-A1/A2, first-, and second-order branch of placental vessels; HPV-A3, placental micro-vessels; SNP, sodium nitroprusside; 5-HT, serotonin. Error bars denote s.e.m. *, P<0.05; **, P<0.01; ***, P<0.001. N, number of participants; n, number of rings.

Basal vascular tension was only slightly decreased by SNP in human umbilical vein and artery, however, SNP showed significant dilation on basal tension in human placental vessels (Figure 3A-3E, Supplementary Table 4 online). Compared with human umbilical vein/artery, serotonin (5-HT)-stimulated vascular constriction was significantly weaker in human placental vessels (Figure 3D and 3E). The similar results of SNP were also shown in sheep placental vessels and non-placental vessels, including sheep carotid artery and renal artery, rat mesenteric artery and thoracic aorta (Figure 3A, and Supplementary Figure 3 online).

### SNP-induced relaxation in preeclamptic placental vessels

**Figure 4:**
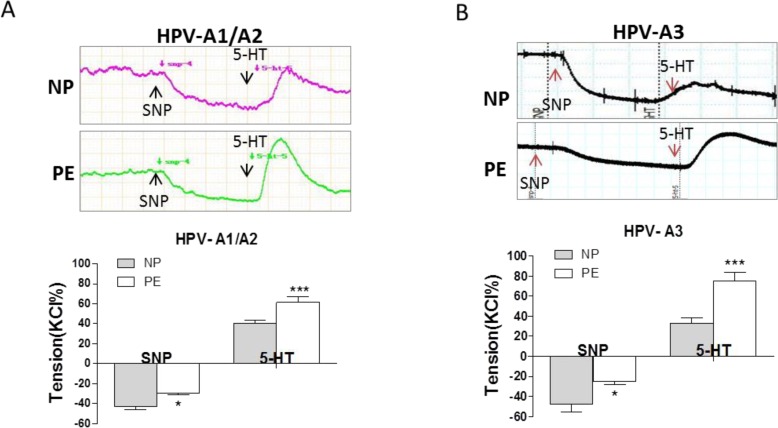
SNP-induced relaxation in preeclamptic placental vessels (**A**), and (**B**), SNP reduced vessel basal tension, followed by 5-HT-stimulated maximum vasoconstriction between normal pregnancies and preeclampsia (NP *vs.* PE) in HPV-A1/A2 (N=31, n=76 for NP; N=23, n=59 for PE) and HPV-A3 (N=35, n=41 for NP; N=36, n=38 for PE). HPV-A1/A2, first-, and second-order branch of placental vessels; HPV-A3, placental micro-vessels; SNP, sodium nitroprusside; 5-HT, serotonin. Error bars denote s.e.m. *, P<0.05; **, P<0.01; ***, P<0.001. N, number of participants; n, number of rings.

Compared to NP, SNP induced vascular relaxation was significantly weaker, whereas, 5-HT-induced maximum vascular tension was significantly higher in PE placental vessels following the pre-treatment of SNP (Figure 4A and 4B, Supplementary Table 5 online).

### Soluble guanylyl cyclase (sGC)/cyclic guanosine monophosphate (cGMP) and K^+^ currents

**Figure 5:**
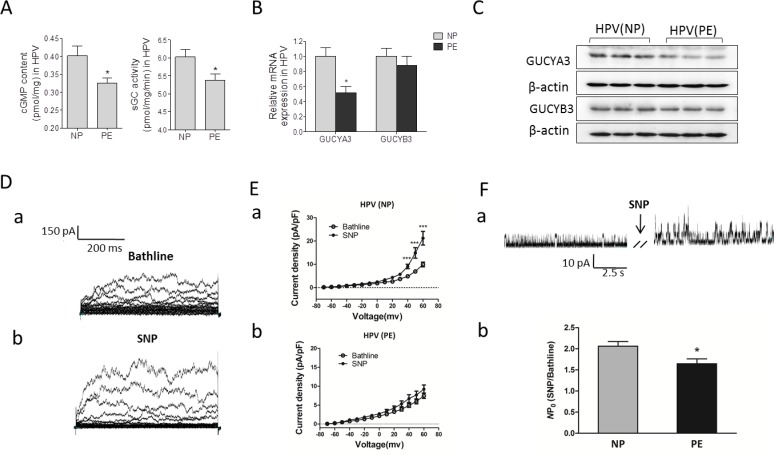
cGMP/sGC pathway and K currents (**A**)-(**C**), cGMP production, sGC activity, and sGC expression in human placental vessel from NP and PE (N=14 for NP; N=15 for PE). (**D**), representative images of whole-cell K^+^ currents before (a) and after (b) application of SNP. (**E**), the effect of SNP on current density in myocytes from HPV (a, n=21 cells/14 women; b, n=19 cells/14 patients). (**F**), (a) representative images of BKCa currents in myocytes before and after application of SNP. (b), the fold change in the *NPo* of BKCa channels after application of SNP in myocytes from HPV (n=15 cells/12 women; n=13 cells/9 patients). NP, normal pregnancy; PE, preeclampsia; cGMP, cyclic guanosine monophosphate; SNP, sodium nitroprusside; HPV, human placental vessel; sGC, soluble guanylyl cyclase; BKCa, Large conductance Ca2+-activated K+ channels; NPo, the open probability. Error bars denote s.e.m. *, P<0.05; ***, P<0.001. N, number of participants.

To determine mechanisms for the decreased effects of SNP in PE placental vessels, we tested cGMP production, sGC activity, and expression of sGC, including α (CUCYA3) and β (CUCYB3) subunits in NP and PE group. cGMP production and sGC activity were significantly decreased in PE placental vessels (Figure 5A). The mRNA and protein levels of CUCYA3, not CUCYB3, were markedly lower in PE placental vessels than that in NP (Figure 5B and 5C). Application of SNP (10^−5^ mol/L) induced a significant increase in whole-cell K^+^ currents (Figure 5D, Supplementary Figure 4a online). The effect of SNP on K^+^ currents in placental vascular smooth muscle cells (VSMCs) from preeclamptic pregnancies was weaker than that from NP (Figure 5E). Single-channel currents of large-Ca^2+^-activated K^+^ channels (BKCa) were significantly enhanced by SNP in both NP and PE group. SNP evoked a 2.1-fold increase in *NPo* (the open probability) of BKCa in NP group, while in PE group, SNP evoked only 1.6-fold increase (Figure 5F). In addition, the whole cell K^+^ currents of mesenteric artery smooth muscle cells were significantly increased by SNP from 30 mV to 60 mV (Supplementary Figure 4b online).

## DISCUSSION

This study focused on placental endothelial functions based on nitric oxide systems, since they are major force for vascular relaxation [11, 12]. Acetylcholine, a classic chemical for vasorelaxation, can dilate almost all kinds of normal intact blood vessels by promoting the release of vasodilators, such as nitric oxide [13]. In the present study, Ach reliably produced vasorelaxation in various non-placental arteries as reported [13–15]. However, in the placenta, no matter large or micro-vessels, Ach showed no relaxation effects on 5-HT- or AII-increased vascular tension, regardless of NP and PE group. Meanwhile, L-NAME did not increase AII-generated vasoconstriction in either NP or PE placental vessels, whereas L-NAME could stimulate AII-induced vasoconstriction in non-placental vessels. It was noted that 5-HT- and AII-generated vascular tension in micro-vessels of the human placenta was reduced in PE group. This interesting phenomenon is worth of further investigation. We also tested sheep and rat vessels to avoid concerns over individual variation in human subjects. Sheep placental vessels also showed that Ach did not have relaxation effects, completely different from non-placental vessels in sheep and other animals. Those data indicated that vasorelaxation effects stimulated by endothelial NO systems in isolated placental vessels were very weak. Previous studies reported that histamine and bradykinin might induce endothelium dependent vasodilation in the perfused placenta [16, 17]. Isolated vessels unlike perfused blood vessels buried in organ tissue, are without any influence from surrounding tissue and local nerve after isolation. When a drug is perfused in vessels surrounded by all kinds of tissue, vascular responses could be direct by the drug and subsequent reactions by signals from tissue surrounded. Thus, different responses to applied drugs between isolated vessels and tissue surrounded vessels could be reasonable. If isolated blood vessels, especially small ones as resistance vessels, showed constriction responses to a drug or stimulation *in vitro*, such vessel responses would induce an increasing of blood pressure *in vivo*; If endothelial NO functions were weak in placental vessels *in vitro*, suggesting that placental endothelial NO-dependent vasodilatation could be poor *in vivo*. In addition, L-Arg concentration, NO production, and eNOS enzymatic activity, as well as expressions of NO enzymes (eNOS and iNOS, not nNOS), in human placental tissue were significantly higher than those in placental vessels in both NP and PE group, indicating that the NO-mediated placental vasodilatation was mainly from placental tissue, not placental vessels. These findings demonstrated new evidences: placental vasorelaxation based on its own endothelial NO signaling was very limited or weak, which were very different from non-placental vessels.

This raised serious question regarding the current theory on hypertension in preeclampsia initiated by placental vascular/endothelial dysfunction-induced ischemia. Notably, literatures show: 1) Most previous experiments did placental tissue instead of placental vessels in analysis of moleculars related to NO; 2) It was too easy to assume placental vessels as the same as non-placental ones; 3) Only a very few studies did placental vascular functional experiments, while effects of Ach were confused by significant variation [18–22], included suspicious relaxation [18], no effects [21], and constriction [22]. Such problems might be due to many causes, and two among those are almost inevitable: first, huge variation in human subjects; second, sample size for functional analysis in human could be too small. Therefore, this study not only employed human samples, also used animal models. Moreover, the large number of human samples (217 independent vessel tests, 89 placentas) was used for functional analysis repeated/verified by third parties. At least 90% in 217 independent tests showed none of Ach vasodilatation effects in human placental vessels, which were reliable and repeatable.

An immediate question raised, what plays roles in placental vasorelaxation under the condition of endothelial NO deficiency? Nitric oxide donor SNP could evoke rapid vasodilatation by providing exogenous NO via cGMP-sGC pathway in VSMCs [23]. In the present study, basal vascular tension was slightly decreased by SNP in human umbilical vein and artery (non-placental vessels), however, SNP showed significant dilation on basal tension in human placental vessels. The similar results of SNP were also shown in sheep placental and non-placental vessels. Specifically, in total 86 placentas with 241 vessel tests, 97.1% showed an obvious reduction of baseline tension following application of SNP, while only 5.2% in 115 non-placental vessel tests showed weak baseline changes. In brief, SNP did not cause relaxation at baseline level in various human, sheep and rat non-placental vessels, whereas SNP produced significant drop from baseline tension in human and sheep placental vessels, regardless of small or large vessels. This is first report to demonstrate that exogenous NO could reliably reduce basal tension of placental vessels in human and animals, which was totally different from non-placental vessels.

Why was there huge difference in response to SNP between placental and non-placental vessels? Non-placental vessels have their own endogenous endothelial NO, which could occupy/act on NO binding sites inside VSMCs. When SNP applied, non-placental vessels had already responded to endogenous NO, thus exogenous NO did not have chance for further changes in vascular baseline tension. On the other hand, placental vessels lacks own endothelial NO, vascular basal tension could be reduced by application of exogenous NO. Those findings provided three new evidences and important messages: 1) Further supported that placental vascular endothelium lacks NO systems/functions so that exogenous NO from SNP could reduce vessel baseline tension significantly; 2) NO is still an important player in placental vascular relaxation, however, that NO was not from placental endothelial cells; 3) If NO is a major force for placental vasodilatation like that in non-placental vessels, lack of endothelial NO in the placenta must have its own physiological meanings for placental vascular regulations in evolution, which deserves further investigations. Those findings suggested, for mechanisms in the placenta for initiating hypertension in preeclampsia, the problem should be in smooth muscle cells or somewhere outside vessels, not endothelial cells. Thus, the target in placental vessels for hypertension in preeclampsia should move from the endothelium to VSMCs or other places.

Compared to normal pregnancy, no matter large or small preeclamptic placental vessels, SNP induced vascular relaxation was significantly weak, while 5-HT-stimulated vascular constriction was markedly increased following pre-treatment with SNP. In PE placental tissue, NO precursor and products, as well as NO enzymes, were not significantly lower than those in NP, while SNP-inhibited vascular tension in PE was weaker, indicating somethings inside VSMCs may not respond properly to the exogenous NO. It is known that NO/cGMP signaling plays vital roles in vasorelaxation [23, 24]. As a key protein in NO/cGMP pathway, sGC is composed of four isoforms: α1, α2, β1, and β2 [25]. Endogenous or exogenous NO can activate sGC to produce cGMP in cells [26, 27]. The increased intracellular levels of cGMP can activate cGMP-dependent PKG [27]. PKG induces a series of phosphorylation that ultimately leads to a decrease of contraction via inhibiting Ca^2+^ influx through Ca^2+^-activated K^+^ channels [27, 28]. In the present study, cGMP production, sGC activity, and expression of CUCYA3 were significantly decreased in preeclamptic placental vessels. In addition, patch clamp experiments showed that SNP induced a significant increase in whole-cell K^+^ currents in both non-placental and placental VSMCs, demonstrating that without endothelial cells, isolated VSMCs of human placental vessels could respond to NO like those non-placental VSMCs. Compared the SNP-increased K^+^ currents, especially BK_Ca_ between NP and PE, the former was higher than the latter, which may be associated with the changes in cGMP, sGC, and CUCYA3 in the placental vessels. Those new data demonstrated that cGMP/sGC pathway in placental VSMCs was injured in preeclampsia.

This study demonstrated that placental endothelial cells are not like previously assumed, behave very differently from non-placental vessels, with very weak endothelial NO functions. If disabled vascular relaxation mediated by NO was a reason for placental ischemia in preeclampsia, cellular targets for the problem should be vascular smooth muscle cells or other places outside the vessels instead of the endothelium. This new information is important for reasonable changes in the current theory: placental vessel changes related to the endothelium as a major cause of placental ischemia initiating pregnant hypertension. Our data offer new insights into the development of hypertension in preeclampsia, and new targets for future treatments of the disease.

## MATERIALS AND METHODS

### Human samples

Pregnant women (N=124) with normal pregnancy (N=76) or preeclampsia (N=48) were recruited from the local hospitals, Suzhou, China. Informed consent for the investigations was obtained. This study was approved by Ethics Committee of the First Affiliated Hospital of Soochow University and all experiments were performed in accordance with the Guidelines and Regulations. Characteristics of the participants are in Supplementary Table 1. Umbilical cords and placenta were immediately collected from healthy and PE participants after deliver vaginally. The middle parts of umbilical cords (100-120 mm in length) and the placenta were kept in iced Krebs solution (containing in mmol/L: NaCl 119, NaHCO_3_ 25, glucose 11, KCl 4.7, KH_2_PO_4_ 1.2, MgSO_4_ 1.0, and CaCl_2_ 2.5), and bubbled with 95% O_2_ and 5% CO_2_. The human umbilical vein (HUV) and artery (HUA) and placental vessels (HPV-A1/A2, first-, second-order branch of blood vessels in placenta, mainly the main stem villous arteries; HPV-A3, branch of the main stem villous arteries, micro-vessels with diameter around 150 um) were carefully isolated. All of the human studies conformed to the principles outlined in the Declaration of Helsinki.

### Animal samples

All animals were from the Animal Center of Soochow University. All experimental procedures were approved by the Institutional Committee of Soochow University and in accordance with the Guide for the Care and Use of Laboratory Animals (NIH Publication No. 85–23, 1996 and 2011 version). Pregnant sheep (gestational age, 130-135 days; term, 147±3 days) were housed in a light-controlled room with standard food and water before general anesthesia. Anesthesia was initiated by ketamine hydrochloride (20 mg/kg, intramuscularly), and general anesthesia was maintained with 1 l/min oxygen and 1-2% isoflurane as described [29]. Under anesthesia, cesarean section was performed, umbilical vein and artery, placental vessels and non-placental vessels such as carotid artery, and renal artery were collected from fetal sheep. Sprague-Dawley rats (5 months old) were sacrificed using sodium pentobarbital intraperitoneally (100 mg/kg; Heng Rui Medicine, Jiangsu, China). Mesenteric artery and thoracic aorta were collected for vascular experiments.

### Measurement of vessel tone

Rings of human, sheep, and rat vessels were prepared, and functional vessel experiments were performed as described [30]. Acetylcholine (10^−4^ mol/L) was used for vessel relaxation experiments after application of 5-HT (10^−5^ mol/L) or AII (10^−5^ mol/L). NG-Nitro-L-arginine Methyl Ester (10^−4^ mol/L) was added into the chambers for 30 minutes before application of angiotensin II (10^−11^-10^−5^mol/L). In addition, the vessel rings were pre-treated with sodium nitroprusside (10^−4^ mol/L) before adding 5-HT (10^−5^ mol/L). Most of vascular functional testing, including the testing on Ach and SNP, were repeated and verified under exactly same conditions by third parties (no authors). Vessels rings after experiments were checked under a microscopy for ensuring intact of endothelium. All drugs for the testing were purchased from Sigma (St. Louis, USA).

### Vascular smooth muscle cells and K^+^ Current Recordings

Vessels were dissected using fine forceps, and cut into 1 mm length and maintained in ice-cold Ca^2+^ free-dissociation media containing (mmol/L): NaCl 130.0, KCl 5.6, MgCl_2_ 1.0, NaH_2_PO_4_ 0.44, Na_2_HPO_4_ 0.42, HEPES 10.0, glucose 10.0, pH 7.4), then cut into 1 mm pieces. Myocytes were isolated enzymatically from dissected vessel pieces as described [31]. The cells were bathed with extracellular solution containing (mmol/L): NaCl 137.0, KCl 5.9, MgCl_2_ 1.2, CaCl_2_ 1.8, glucose 10.0, HEPES 10.0, pH 7.4. For whole-cell recording, borosilicate glass electrodes were pulled with a horizontal pipette puller (P-97, Sutter Instrument Co, Novato, CA) and had tip resistances of about 3-5 MΩ when filled with the pipette solution containing (mmol/L) K-aspartate 110.0, KCl 30.0, NaCl 10.0, MgCl_2_ 1.0, EGTA 0.05, and HEPES 10.0 (pH 7.3). The whole-cell patches used for analysis should be with a series resistance <10 MΩ, seal resistances >2 GΩ, leakage current < 50pA. Cells were voltage clamped at −60 mV and step depolarised from −70 mV to +60 mV for 540 ms in 10 mV increments and repolarised to −60 mV. For cell-attached patch, currents were recorded using patch pipettes with about 9-10 MΩ. Recording was conducted under symmetrical K^+^ conditions with pipette solution containing in (mmol/L): KCl 140, CaCl_2_ 0.8, MgCl_2_ 1, HEPES 10, pH 7.4. The single-channel current was recorded at a holding potential at +60mV. Channel activity was expressed as *NPo*. Continuous recordings of no less than 3 min were used for *NPo* analysis. Data were sampled at 20 kHz and filtered at 2 kHz. Current signals were acquisitioned using an Axopatch 700B amplifier with pCLAMP 10.2 Pulse software (Axon Instruments, Foster City, CA).

### Quantitative Real-Time PCR (qRT-PCR) and western blot analysis

Protein and mRNA levels of nNOS, iNOS, eNOS, GUCYA3, and GUCYB3 in human placental vessels and tissue were measured. All primers were listed in Supplementary Table 2. The protein abundance of nNOS, iNOS, eNOS, GUCYA3, and GUCYB3 were assessed by Western blotting normalized to β-actin. Antibodies were purchased from Santa Cruz Biotechnology (Santa Cruz, CA, USA). Western blot and qRT-PCR analyses were performed as described [32, 33].

### Nitric oxide production, eNOS, and sGC activity

Two end products of nitric oxide, nitrite and nitrate, were measured using Nitrate/Nitrite Assay Kit (Beyotime Biotechnology). eNOS enzymatic activity was tested with Nitric Oxide Synthase Assay Kit (HY-2088) according to the manufacturer's protocols (Beijing Huaying Biotechnology Research Institute, China.). Soluble guanylyl cyclase enzyme activity in tissue was assayed by formation of [32P] cGMP from [α-32P] guanosine triphosphate (GTP) at 37°C as described previously [34]. Soluble guanylyl cyclase enzyme activity is expressed as pmol of cGMP produced per minute per milligram of protein (pmol/mg/min) in human placental vessels.

### Radioimmunoassay (RIA)

L-Arg and cGMP content in human placenta was determined using RIA kit (HY-60111 and HY-100781). Radioimmunoassay was performed according to the manufacturer's instructions (Beijing Huaying Biotechnology Research Institute, China.). The sensitivity was 0.01umol/g (L-Arg), and 0.001pmol/mg (cGMP), respectively. The intra-assay and inter-assay CV were 3.0-8.7% and 7.0-9.9%, respectively. The samples and data were handled in a blind manner.

### Data analysis and statistics

Data were expressed as the mean ± SEM. Statistical analysis was performed with t-test or two-way analysis of variance (ANOVA). Concentration-response curves for vascular responses were analyzed by computer-assisted nonlinear regression (Graph Pad Prism software) to fit the data. Statistical significance was accepted at P <0.05.

## SUPPLEMENTARY MATERIAL


