# Supplementary material for: New conception for the development of hypertension in preeclampsia
Source: Oncotarget. 2016 Nov 16;7(48):78387–95. doi: 10.18632/oncotarget.13410 (PMC5346647; doi:10.18632/oncotarget.13410)
Supplement: Supplementary file 1 [file oncotarget-07-78387-s001.pdf]

New conception for the development of hypertension in preeclampsia

Supplementary Material

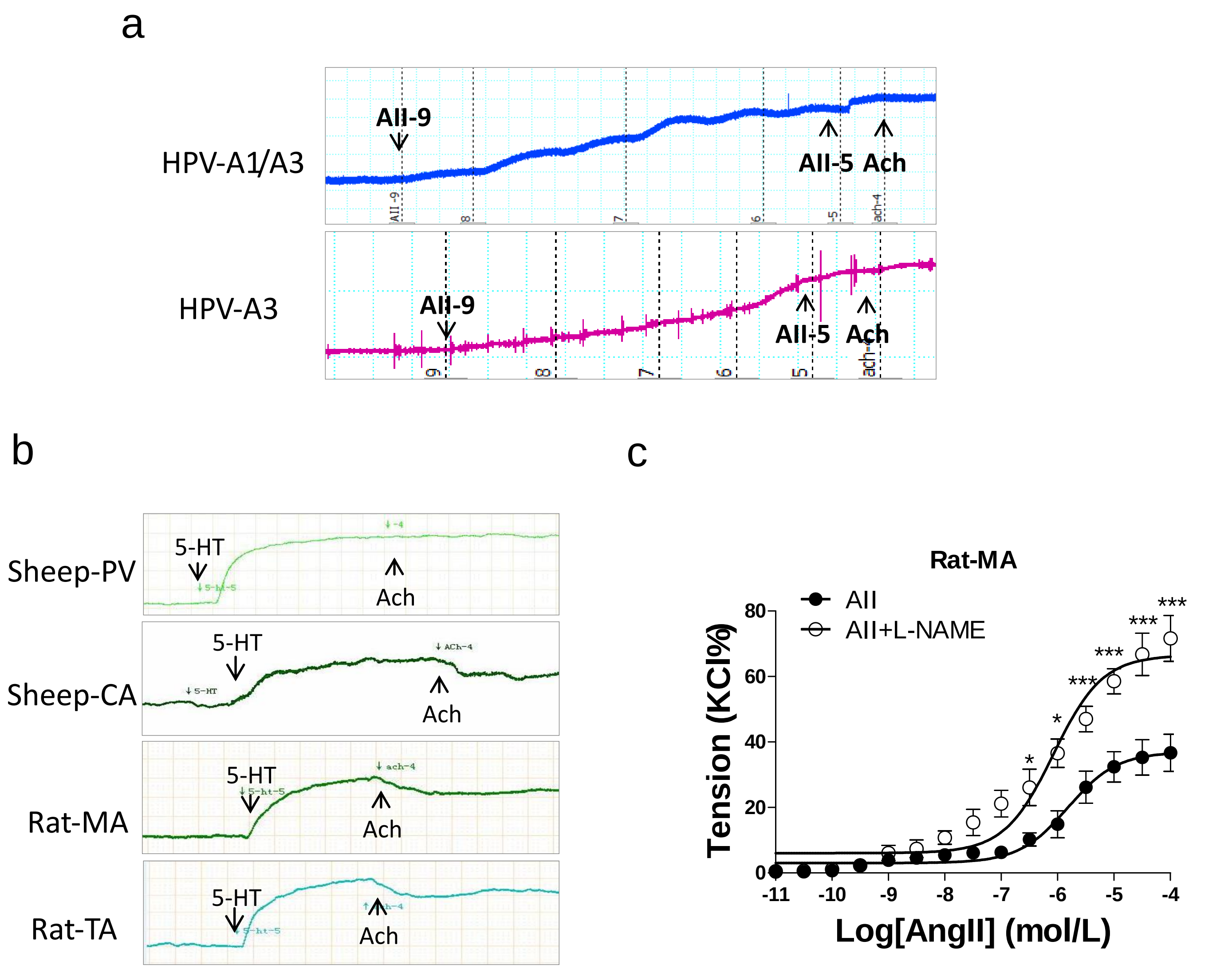

Figure S1. Endothelium-dependent vasodilatation in placental vs. non-placental vessels. (a), representative records of Ach-induced vasodilatation following AII-produced vasoconstriction in human placental vessels. (b), representative images of Ach-induced vasodilatation following 5-HT-produced vasoconstriction in sheep placental vessels (PV), sheep carotid artery (CA), rat mesenteric artery (MA) and thoracic aorta (TA). (c), pre-treated with L-NAME inhibited AII-induced dose-dependent vasoconstriction in rat mesenteric artery (N=12, n=16). SNP, sodium nitroprusside; 5-HT, serotonin; L-NAME, NG-Nitro-L-arginine Methyl Ester; AII or AngII, angiotensin II; Ach, Acetylcholine. Error bars denote s.e.m. *P<0.05; ***P<0.001. N, Rat number; n, vessel ring number.

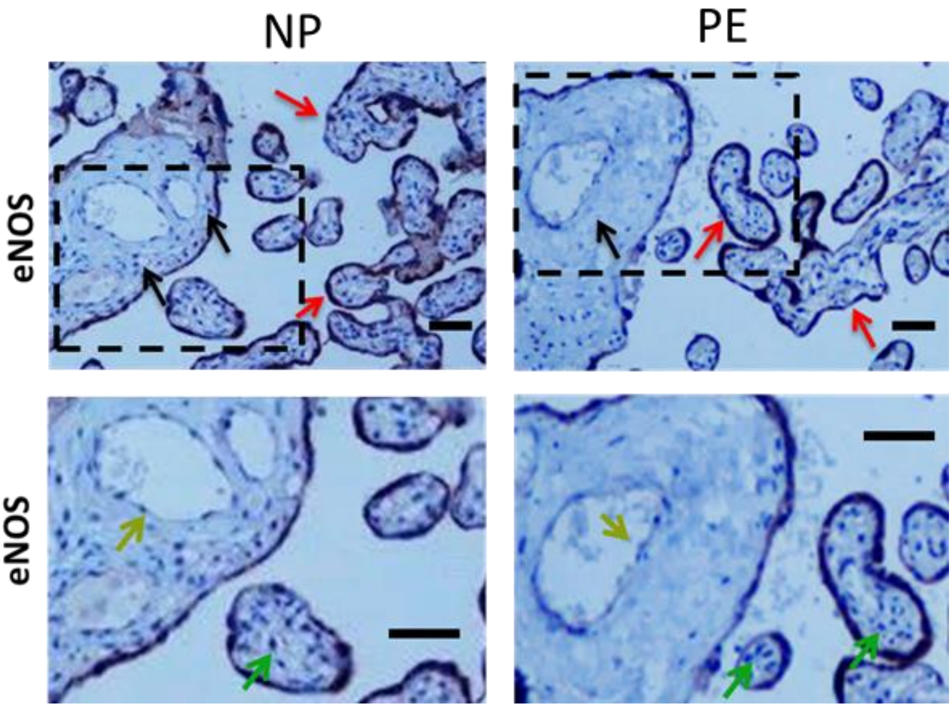

Figure S2. eNOS staining were more intensive in the surrounding tissue than that at the inside layer of placental vessel wall, regardless of normal pregnancy (NP) or preeclampsia (PE). Black arrows: Wall of placental vessels; Red arrows: placental tissue surrounding blood vessels; Yellow arrows: positive eNOS in placental vessels; Green arrows: positive eNOS in placental tissue.

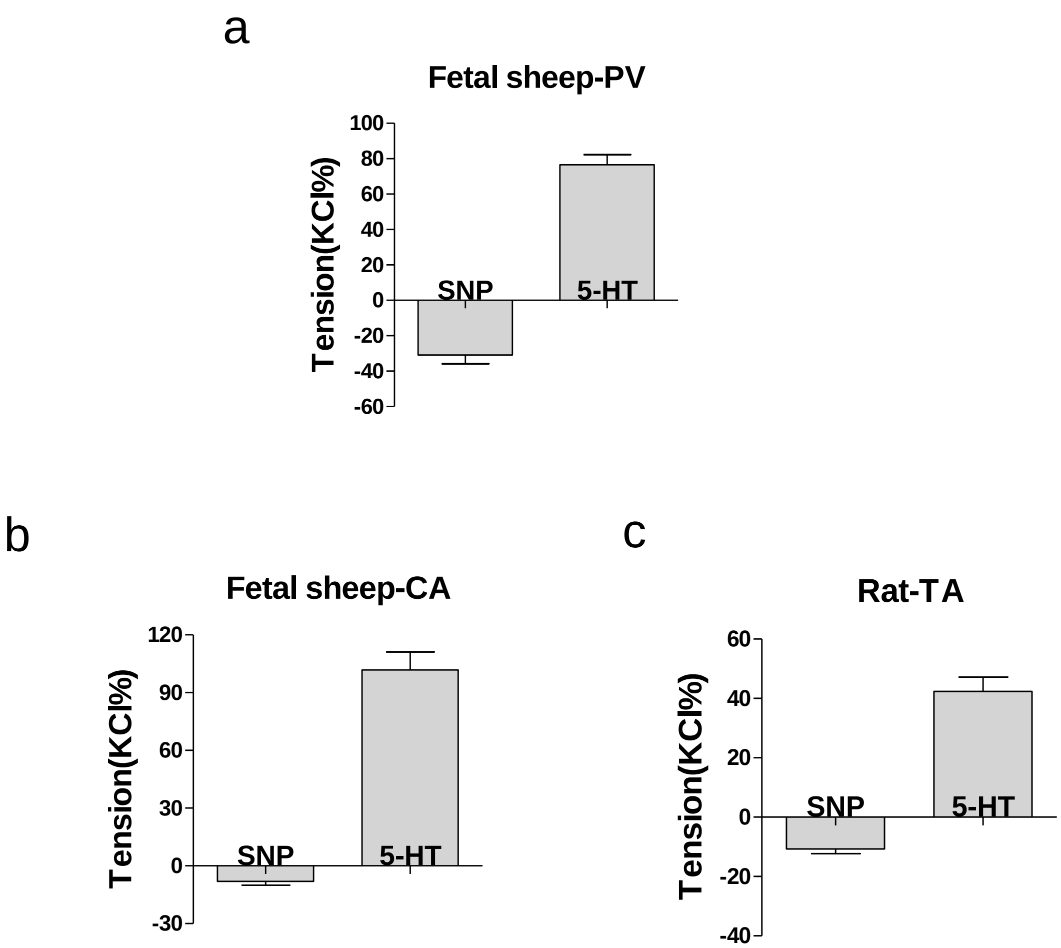

Figure S3. SNP reduced vessel baseline tension, followed by 5-HT-stimulated maximum vasoconstriction in the placental and non-placental vessels. (a), and (b), fetal sheep placental vessels (PV)(N=8, n=17) and carotid arteries (CA)(N=7, n=11). (c), rat thoracic aorta (TA) (N=6, n=19). SNP, sodium nitroprusside; 5-HT, serotonin. Error bars denote s.e.m. N, Sheep or Rat number; n, vessel ring number.

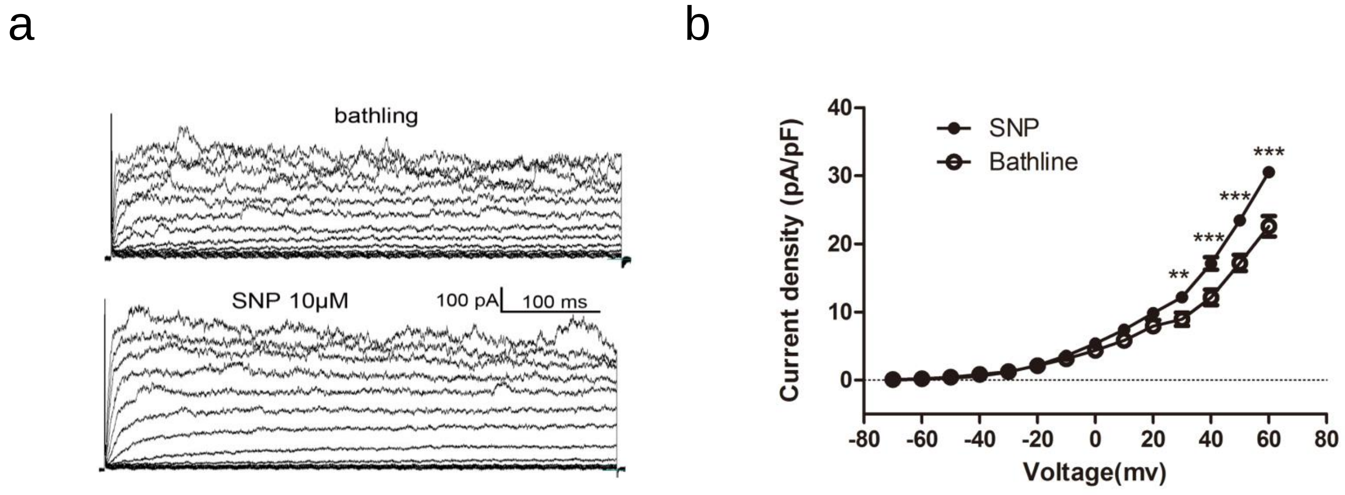

Figure S4. The effect of SNP on K^+ currents in the myocytes of rat mesenteric arteries. (a), representative currents measured in whole-cell mode before (upper panel) and after (lower panel) application of SNP. (b), current density versus voltage plot in the absence and presence (n=20 cells/10 rats) of SNP in the myocytes from rat mesenteric arteries. SNP, sodium nitroprusside. Error bars denote s.e.m. **P<0.01; ***P<0.001.

Table S1. Basic characteristics of PE cases and normotensive controls. S/D ratio, ratio of systolic and diastolic blood flow in the umbilical artery. The date was expressed as mean±SD. *P<0.05; **P<0.01. NP, normal pregnancy; PE, preeclampsia.

Characteristics	NP	PE
Number of subjects	76	48
Maternal age(y)	29.40±4.20	28.90±4.40
Gestational age (wk)	37.90±2.10	34.30±5.40*
Birth weight (kg)	3.23±0.53	2.75±0.97*
Systolic BP (mm Hg)	116.6±9.10	157.5±16.10**
Diastolic BP (mm Hg)	78.40±7.31	107.2±14.40**
Proteinuria (g/24h)	0.16±0.05	7.93±4.86**
BMI--Body Mass Index	26.92±2.57	28.97±3.18
S/D ratio	2.18±0.24	3.49±0.46*

Table S2. List of oligonucleotide primers used.

qRT-PCR primers	Sequence	
Gene Name	5′-3′	3′-5′
Human-nNOS	TCACGGCTCAGTCCCTACATA	TGCGGATCAGATCTGAGGCAT
Human-iNOS	CAGTAGAGAACATCTG AGCTC	GACTCATTCTGCTGCTTGCTGA
Human-eNOS	AGACTCTTCCGGAAGGCTTTTGA	TTCCTGGAAGTTCTCACCTCT
Human-GUCYA3	TGCCCATCTGTCAAGACATTCC	GTGCTTTGCCAATGCTTTGC
Human-GUCYB3	TCTCAATGCTGGAGAAATCCTCC	GTAGATGGTAGCAAGGTGGTC

Table S3. Percentage of vessel rings for Ach-mediated relaxation responses following 5-HT-produced vasoconstriction in human placental vessels. Ach, acetylcholine; NP, normal pregnancy; PE, preeclampsia. N, placenta number; n, rings number.

	N	n	no response(n)
NP	52	124	113(91.13%)
PE	37	93	84(90.32%)

Table S4. Percentage of vessel rings for SNP-reduced baseline tension in placental and non-placental vessels. Placental vessels included human placental vessels (N=76, n=204) and sheep placental vessels (N=10, n=37). Non-placental vessels included fetal sheep carotid artery (N=9, n=28) and renal artery (N=9, n=33), rat thoracic aorta (N=8, n=37) and mesenteric artery (N=10, n=17). SNP, sodium nitroprusside. N, Placenta number; n, vessel ring number.

	**N**	**n**	**Reduced basal tension(n)**
**Placental vessels**	86	241	234(97.09%)
**Non-placental vessels**	36	115	6(5.22%)

Table S5. Percentage of vessel rings for 5-HT-induced vasoconstriction following application of SNP -reduced vascular tension in human placental vessels. HPV-A1/A2, first-, and second-order branch of placental vessels; HPV-A3, micro-vessels; 5-HT, serotonin; NP, normal pregnancy; PE, preeclampsia. N, Placenta number; n, rings number.

		**N**	**n**	5-HT/KCl (<15%)	5-HT/KCl (15%-50%)	5-HT/KCl(>50%)
**HPV-A1/A2**	NP	31	76	23(30.26%)	28(36.84%)	25(32.89%)
	PE	23	59	8(13.56%)	17(28.81%)	34(57.63%)
**HPV-A3**	NP	35	41	11(26.83%)	16(39.02%)	14(34.15%)
	PE	36	38	5(13.16%)	13(34.21%)	20(52.63%)
